# The Surgical Management of a Radicular Cyst in the Maxillary Anterior Region - A Case Report

**DOI:** 10.7759/cureus.59216

**Published:** 2024-04-28

**Authors:** Tikeshwari Gurav, Priyanka Sontakke, Aman Thakare, Amit Reche, Dhruvi Solanki

**Affiliations:** 1 Department of Public Health Dentistry, Sharad Pawar Dental College and Hospital, Datta Meghe Institute of Higher Education and Research, Wardha, IND; 2 Department of Oral Medicine and Radiology, Sarswati Dhanwantari Dental College and Hospital,Parbhani, Wardha, IND; 3 Department of Public Health Dentistry, Sharad Pawar Dental College and Hospital, Datta Meghe Institute of Higher Education and Research, wardha, IND; 4 Department of Pedodontics and Preventive Dentistry, Sharad Pawar Dental College, Datta Meghe Institute of Higher Education and Research, Wardha, IND

**Keywords:** odontogenic cyst, maxilla, enucleation, periapical infection, radicular cyst

## Abstract

Radicular cysts are the most common forms of cysts in the jaws. They develop from epithelial residues in the periodontal ligament in response to periapical infection following pulpal necrosis. This condition is typically asymptomatic and mostly affects the tooth's apices. It primarily affects non-vital teeth and is characterized by inflammation. Cyst development is the final stage of the inflammatory process after a periapical infection; hence, it often occurs later in life. A cyst in the maxilla can occasionally spread across the maxillary sinus. Radicular cysts can be treated with surgical endodontics, the removal of the problematic tooth, enucleation with primary closure, or marsupialization and enucleation. This case report discusses a successful surgical therapy for an infected radicular cyst.

## Introduction

A cyst is a pathological cavity that may or may not be lined by epithelium and is always filled with fluid or semifluid material but not pus. It is a space-occupying lesion with a fibrous connective tissue wall surrounding a core hollow known as the lumen. On the inner side of the wall, where there is a lining of the epithelium, it is mainly stratified squamous epithelium. Aetiology determines whether they are developmental or odontogenic. The maxillofacial area has a higher incidence of cysts [1,2].

Radicular cysts are classified as odontogenic cysts by the WHO (2017) [1]. They are commonly observed in the apex of teeth with diseased or necrotic pulp. However, they can also be found on the lateral portions of the roots as a result of accessory root canals. Radicular cysts are the most prevalent (52%-68%) cystic lesions in the jaws [3]. It is more common in the maxillary anterior region than in the mandible. Aside from the pathogenic tooth, adjacent healthy teeth in the cystic capsule are known as "included teeth" or "involved teeth "[4].

Radicular cysts of small size are usually treated conservatively, whereas larger ones require surgical enucleation. They are usually painless unless secondarily infected. To treat a cyst, the affected teeth may be indicated for extraction if it has a poor prognosis. To prevent the recurrence of the lesion, all epithelial remnants must be removed from the cystic wall. This case report discusses in detail a case of radicular cyst involving the maxillary anterior quadrant treated surgically by enucleating the lesion.

## Case presentation

The 53-year-old female patient reported to the Department of Oral and Maxillofacial Surgery with a chief complaint of pain localized to the upper front tooth region of the jaw. She described the pain as dull and throbbing in nature. The pain had been present for the past week. The patient did not report any history of trauma to the affected area. There was no associated swelling, fever, or discharge. She reported that the pain worsened while chewing and applying pressure on the affected tooth. The patient had tried over-the-counter pain medications, which provided temporary relief but did not alleviate the pain completely. She denied any other dental or oral health issues in the past. The pain did not radiate to any other part of the face or head, and there were no aggravating or relieving factors reported by the patient.

An intraoral clinical examination revealed missing 26, 35, 36, 45 and 46. Both upper central and lateral incisors showed tenderness on percussion. Electric and thermal pulp vitality tests revealed a negative response. The patient had overall poor oral hygiene, with bleeding upon probing. Gingival recession was present concerning lower anterior teeth. Table 1 shows the endodontic status of the maxillary anterior teeth. Table 1 shows the endodontic status of the maxillary anterior teeth.

**Table 1:** Endodontic evaluation

Tooth No	Length	Roots	Canals	Periapical region
11	20.84mm approx	Single root, straight and completely formed. Apical root resorption.	The canal is thin & patent till the apical region.	Lesion/Pathology evident.
12	19.47mm approx	Single root, straight and completely formed till apex	The canal is thinned & patent till the apex.	Lesion/Pathology evident.
13	23.81mm approx	Single root, straight and completely formed till apex.	The canal is thinned & patent till the apex.	Lesion/Pathology evident.
21	21.26mm approx	Single root, straight and completely formed. Apical root resorption.	The canal is thin & patent till the apical region.	Lesion/Pathology evident
22	19.58mm approx	Single root, straight and completely formed till apex.	The canal is thinned & patent till the apex.	Lesion/Pathology evident
23	22.40mm approx	Single root, straight and completely formed till apex.	The canal is thinned & patent till the apex.	Lesion/Pathology evident

**Figure 1:**
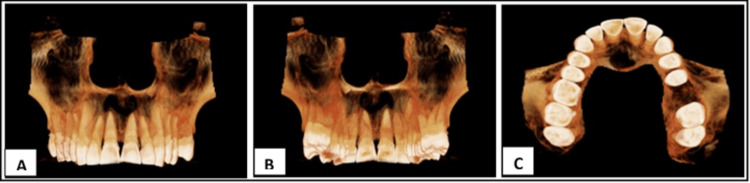
Maxillary CBCT imaging showing radicular cyst involving anterior quadrant: A) buccal view; B) palatal view; C) occlusal view CBCT - cone beam computed tomography

**Figure 2:**
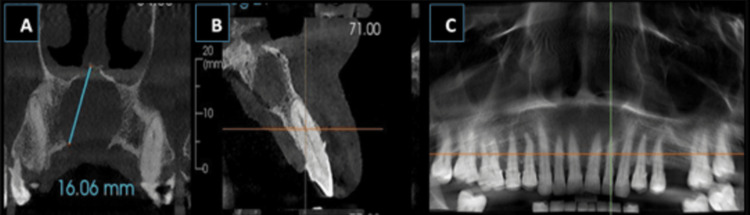
CBCT images showing: A) cystic extension at the maxillary midline; B) palatal relation of the cyst with the tooth root; C) cystic extension to the nasal floor and its involvement CBCT - cone beam computed tomography

Cone beam computed tomography was indicated with the upper quadrant, which revealed an unilocular, well-defined, iso-hypodense lesion with corticated borders. The lesion was oval in shape, extending from the labial cortical plates involving the mid-alveolar cortex region to the palatal cortical plates (bucco-palatally) and involving the nasal floor up to the periapex and apical region of 11, 12, 13, 21, and 22 region (supero-inferiorly). The lesion crossed the midline from 13 to 22 and measured approximately 15.7mm (mesiodistally) x 13.51mm (bucco-palatally) x 16.06mm (height). Thinning and breach in the labial cortical plates were noted in the 11, 12, 21, and 22 region. A breach in the palatal cortical plates and the nasal floor was also evident, along with loss of periodontal ligament space concerning 11 to 23 and evidence of apical root resorption with 11 and 21 (Figure 1 and Figure 2). Apart from these, generalized mild-moderate alveolar bone loss was evident. The internal structure appears to be completely hypodense with a complete loss of trabecular pattern internally. Loss of sclerotic border was evident with the naso-palatine canal.

Considering the radiographic findings that was suggestive of osteolytic lesion with 13-23 region, diagnosis of infected radicular cyst with anterior region was given.

Treatment

Based on all the examinations, it was planned to surgically enucleate the teeth and do root canal treatment for 13, 12, 11, 21, 22, and 23. After informing the patient about the treatment plan and obtaining proper consent, the canals of the concerned teeth (13-23) were opened, debrided, and dressed with calcium hydroxide. This was followed by surgical enucleation of the cyst via apicoectomy and retrograde filling of the afflicted teeth.

**Figure 3:**
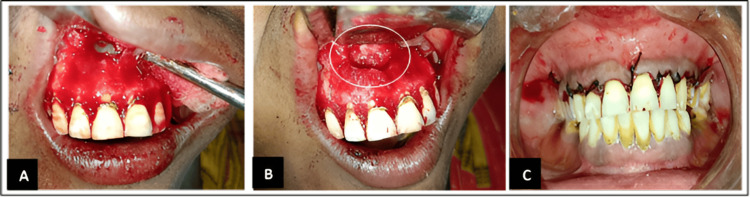
Intra-operative pictures showing A) alevation of the full-thickness buccal mucoperiosteal flap; B) cortical bone window; C) placement of sutures

After administering local anesthesia (LA), a full-thickness mucoperiosteal flap was reflected and irrigated with normal saline, as shown in Figure 1. A crevicular incision was made, followed by elevation of the palatal full-thickness mucoperiosteal flap to reveal the lesion. The cystic lesion was exposed by removing the underlying bone with a bur under extensive irrigation to create a window for access. The cortical bone window was then widened, exposing underlying disease and allowing for adequate curettage. The lesion was carefully separated from the nasopalatine nerve and artery and was completely removed using curettage and enucleation. The curettage of the cyst was completed, and granulation tissue was excised. This was followed by thorough irrigation with betadine and normal saline, and flap closure was achieved with a 3-0 silk suture following hemostasis, as shown in Figure 3.

The excised tissue was then sent for histopathological investigation, which confirmed the diagnosis of infected radicular cysts. The patient was given post-operative instructions and continued to take antibiotics (amoxicillin) and analgesics (keterol). The patient was called back for post-operative evaluation every day, seven days, six months, and a year.

## Discussion

Periapical cysts are inflammatory jaw cysts that form at the apex of broken teeth with necrotic pulp. They are classified as bay or apical according to how the root canal connects to the epithelial cavity. A bay cyst is a cavity of cysts with epithelial linings that opens to the root canal. Because it resembles the marginal periodontal pocket, it is commonly known as a "periapical pocket cyst". Apical cysts have full epithelialization but do not open into the apical foramen or root canal. Currently, it is known as a radicular cyst or a true cyst [5, 6].

Radicular cysts account for around 52%-68% of jaw cysts overall. They frequently appear in the maxillary anterior area during the third and fifth decades of life, with men being more likely to develop them [7]. Inflammation ensues following the necrosis of dental pulp either due to caries or trauma, which eventually leads to granuloma, Malassez cell rest activation, and cyst formation if left untreated for a very long period. Cysts form when epithelial residues in the periodontal ligament become inflamed. Radicular cysts are often thought to develop in three stages: initiation, cyst development, and cyst growth [8,9]. The porous texture of the maxillary bone, along with a reluctance to remove anterior teeth, may facilitate cyst formation. Radicular cysts start as a bony, hard swelling, but as they expand, the surrounding bone may decrease despite early sub-periosteal bone deposition. Swelling may demonstrate springiness or 'eggshell cracking' as bone resorption advances [7]. The epithelium encircling the cyst can come from several sources, including Malassez cell rest, crevicular epithelium, sinus lining, or fistulous tract epithelium [10]. This lesion's usual consequences include root resorption and displacement of surrounding teeth. Radicular cysts may cause jaw swelling, pain, and tooth loosening [11].

Treatment for radicular cysts is determined by the lesion's location, proximity to essential structures, clinical presentation, and the patient's overall health. Radicular cysts can be treated using both surgical and non-surgical techniques. Surgical procedures involve enucleation and marsupialization. Enucleation is commonly done for small cysts that do not impact vital structures. The combined method lowers morbidity and accelerates the repair of the lesion [12]. Non-surgical therapy options include conservative endodontic treatment, decompression, aspiration, irrigation, calcium hydroxide, lesion sterilization and repair, and the Apexum technique. Simvastatin and epigallocatechin are also being investigated [13].

The cystic fluid is used to identify odontogenic cysts. The cystic composition could range from a translucent yellow fluid to a solid, cheese-shaped mass. The total protein content normally varies from 5 to 11 g/100 ml. Other odontogenic cysts, including keratocysts and dentigerous cysts, have lower protein concentrations than this [14]. The cavity is surrounded by nonkeratinized stratified squamous epithelium, which may be discontinuous in areas with strong inflammatory cell infiltration. During the early phases, epithelial lining cells may multiply and form an arcading pattern, which is accompanied by ongoing inflammatory infiltration. In rare cases, small quantities of keratin may be discovered [1].

Rushton bodies, often known as hyaline, are odontogenic epithelial components that resemble cuticular or keratin and are frequently seen histologically in cystic lesions. Another idea holds that they are secretions from activated epithelial cells that eventually calcify. Vascular thrombosis is also present, caused by blood vessels being trapped inside the epithelium [15]. It might be caused by elastotic degeneration or a cellular reaction to extravasated serum [16]. It could be caused by elastotic degeneration or a cellular response to extravasated serum [17].

In this case, the lesion was developed as a result of trauma. Radicular cysts have a poor prognosis due to malignant alteration of the lining epithelial cells. A study found that squamous odontogenic tumor-like proliferations can develop inside the radicular cyst lining [18]. Radicular cysts in the maxillary area were the most typical sites for such change [19]. Radicular cysts should be treated promptly to prevent problems.

Clinical implications

Radicular cysts, being a less common variety of odontogenic cysts, carry a greater significance in diagnosis. Diagnosis should be made more precisely as most of these cases are diagnosed while the patient is either partially or completely unaware of it. So, the diagnosis followed by patient counseling carries significance as to make them understand the treatment plan, which usually involves surgery. The surgery, however unappealing, is the treatment of choice, and the patient should be made aware of the same. Post-surgery, the patient should be counseled on how to manage the wound, and periodic follow-ups should be made to lower the post-surgical complications. 

## Conclusions

Radicular cysts are commonly observed in the oral cavity. However, it typically passes unnoticed and rarely reaches palpable proportions. The treatment choices for cysts vary based on their size and location. Endodontic therapy with surgical enucleation is recommended for long-standing chronic lesions; however, nonsurgical care for minor lesions can also be advocated.
